# Adsorption of amino acids by fullerenes and fullerene nanowhiskers

**DOI:** 10.1088/1468-6996/16/6/065005

**Published:** 2015-11-27

**Authors:** Hideo Hashizume, Chika Hirata, Kazuko Fujii, Kun’ichi Miyazawa

**Affiliations:** National Institute for Materials Science, 1-1 Namiki, Tsukuba, 305-0044, Japan

**Keywords:** adsorption, amino acid, fullerene, fullerene nanowhisker

## Abstract

We have investigated the adsorption of some amino acids and an oligopeptide by fullerene (C_60_) and fullerene nanowhiskers (FNWs). C_60_ and FNWs hardly adsorbed amino acids. Most of the amino acids used have a hydrophobic side chain. Ala and Val, with an alkyl chain, were not adsorbed by the C_60_ or FNWs. Trp, Phe and Pro, with a cyclic structure, were not adsorbed by them either. The aromatic group of C_60_ did not interact with the side chain. The carboxyl or amino group, with the frame structure of an amino acid, has a positive or negative charge in solution. It is likely that the C_60_ and FNWs would not prefer the charged carboxyl or amino group. Tri-Ala was adsorbed slightly by the C_60_ and FNWs. The carboxyl or amino group is not close to the center of the methyl group of Tri-Ala. One of the methyl groups in Tri-Ala would interact with the aromatic structure of the C_60_ and FNWs. We compared our results with the theoretical interaction of 20 bio-amino acids with C_60_. The theoretical simulations showed the bonding distance between C_60_ and an amino acid and the dissociation energy. The dissociation energy was shown to increase in the order, Val < Phe < Pro < Asp < Ala < Trp < Tyr < Arg < Leu. However, the simulation was not consistent with our experimental results. The adsorption of albumin (a protein) by C_60_ showed the effect on the side chains of Try and Trp. The structure of albumin was changed a little by C_60_. In our study Try and Tyr were hardly adsorbed by C_60_ and FNWs. These amino acids did not show a different adsorption behavior compared with other amino acids. The adsorptive behavior of mono-amino acids might be different from that of polypeptides.

## 1. Introduction

Over the last few decades, various nanomaterials have been developed and utilized in various areas, such as the medical, pharmaceutical, paint and cosmetics industries. Their effects on cells, skin, lungs and other organs have been studied and discussed [1]. Some nanomaterials were found to be very harmful to the human body [2, 3]. The toxicity of nanocarbons like graphene, fullerene and carbon nanotubes has also been investigated.

Nanocarbons like graphene, fullerene and carbon nanotubes have also been investigated for the risks they pose to our body. Carbon nanotubes pose a high risk to our lungs because they are needle shaped, like asbestos [4]. The effect of nanocarbons entering cells is not yet well understood. Recently an interaction between nanocarbons and biomolecules has been studied. The adsorption of amino acids by fullerenes C_60_ and C_80_ was investigated using a computer simulation and theoretical calculation [5, 6]. And the adsorption of amino acids by a single-wall carbon nanotube including a metal cation in the tube was also investigated by theoretical simulation. The interaction between nanocarbons and amino acids has not yet been investigated experimentally. The interaction between lipids or nucleic bases and nanocarbon materials has hardly been investigated theoretically or experimentally [7, 8]. It is necessary to know about the interaction of carbon materials with proteins in order to study the relationship between a carbon nanomaterial and a protein or an amino acid. For example, the interactions between mesoporous carbons and proteins have been well investigated by Vinu *et al* [9, 10].

Kroto *et al* introduced fullerene (C_60_) [11].  Since then, fullerene has been improved so as to be used for various purposes. The relationship between C_60_ and/or modified C_60_ and organic polymers was investigated by Babu *et al* [12]. Various formed C_60_ nanomaterials have been designed such as nanowhiskers, nanotubes, and a sheet of C_60_ aggregate [12–14]. C_60_ nanowhiskers (FNWs) were designed and created by Miyazawa *et al* [15]. The diameter of the FNWs is around 100 nm and length is from several hundreds of nm to several mm. The diameter and length are controllable by changing the experimental conditions [16, 17]. The relationships between the arrayed FNWs and cells have been investigated by Minami *et al* and Krishnan *et al* [18, 19]. They showed that cells enlarged towards the direction of the ordered FNWs. Unfortunately they did not report on the toxicity of the FNWs to cells.

Nanomaterials can cause damage to a cell or a gene; they might intrude into the cell from a membrane protein, by an affinity of lipids, or by penetration through a membrane [20, 21]. To clarify the interactions between a membrane protein and C_60_ or FNWs, we have experimentally investigated the adsorption of amino acids and an oligopeptide constituting a protein by C_60_ or FNWs, and compared the results with previous theoretical predictions.

## 2. Experimental

### 2.1. Materials

C_60_ (99.5%) was purchased from MTR Ltd, USA. D-tyrosin (Tyr), D-triptphane (Trp), D-phenylalanine (Phe), D-proline (Pro), D-leusine (Leu), D-aspartic acid (Asp), D-valine (Val) and D-argenine (Arg) were obtained from Wako Chemical, Japan and D-alanine, (Ala) D-alaninyl-D-anlanyl-D-alanine (Tri-Ala) were purchased from Sigma USA. We used the D-enanthiomer of the amino acids to minimize external contamination and detrimental effects on microorganisms

### 2.2. Adsorption

Solutions of amino acids were prepared with concentrations of up to 2 mmol dm^−3^. In the case of Trp, since solubility is relatively low, we used concentration prepared solution of up to 1 mmol dm^−3^. The solution was adjusted around the isoelectric point of each amino acid to use 0.1 mol dm^−3^ HCl and/or Na(OH). Several diluted solutions were used in an adsorption treatment.

**Figure 1.**
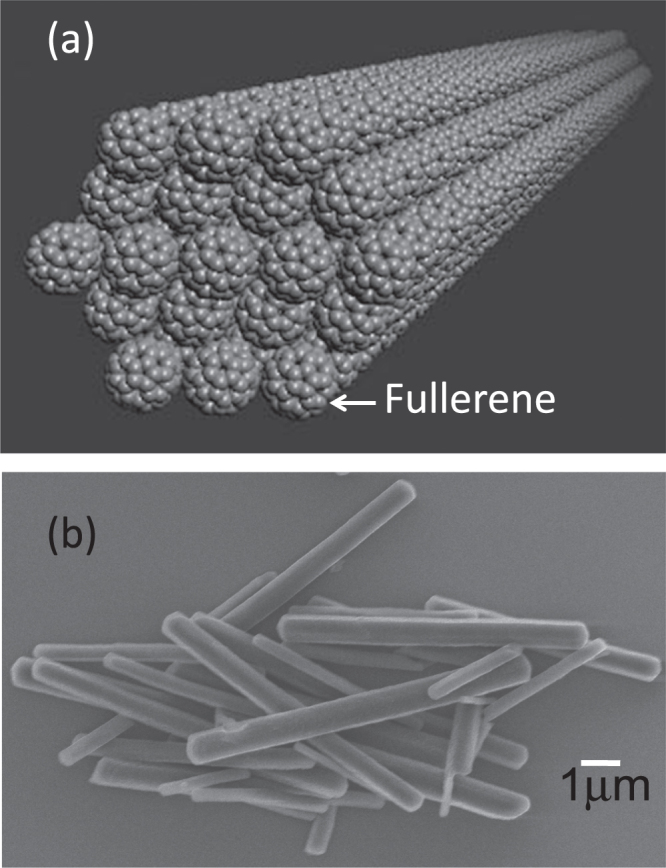
Schematic (a) and SEM image (b) of FNWs.

The FNWs were made according to Miayzawa *et al* [22]. C_60_ molecules formed aggregates with diameters of approximately 1.8 *μ*m. The FNWs were 645 ± 170 nm wide and 6.6 ± 2.5 *μ*m long. A scanning electron miscroscopy (SEM) image and an illustration of the FNWs are shown in figure 1.

Around 0.02 g of C_60_ or FNWs was weighed by an analytical scale, as an adsorbent. The correct weight was used in estimating adsorption. The weighed adsorbent and 6.5 cm^3^ of an amino acid solution were put in a sample bottle with a stopper. After the stopper was tightly closed, the sample bottle was shaken for 3 h by a rotational shaker (RKVSE, ATR, USA). The rotational speed was 60 rpm. After shaking the sample bottle, the equilibrium pH was measured (pH meter TPX-999, Toko chemical Laboratories Co. LTD). PHs of the suspension after the adsorption treatments were different from the pH of the initial solution. The pHs of the suspension are shown in table 1. The suspension was filtered using a 0.2 *μ*m disposal filter. The initial solution and treated solution were measured by a total organic carbon analyzer (TOC-5000A, Shimadzu, Japan). The TOC measured the infrared absorption of a C = O bond. The solution is sprayed into a silica tube with catalysis in a furnace at 680 °C in which the highly purified air is flown. Organic compounds are burned, the CO_2_ gas produced was carried to the CO_2_ detector with the flowing gas, and the concentration of carbon dioxide was measured. We used glycine as a standard material. One sample was measured seven or eight times. We chose five to eight raw data for which the CV value was the smallest or less than 0.01. A CV value is the ratio of (standard deviation)/(average value). Concentrations of the initial and treated solution were obtained by the average of the chosen data. Adsorption was evaluated by *A* = *V*(C-C_0_)/*W*, where *A* is adsorption, *V* is volume of solution, *C* is the concentration of the treated solution, C_0_ is the concentration of the initial solution and *W* is the weight of adsorbent.

**Table 1.** pHs of suspensions after the adsorption treatment.

Amino acid	pH	Amino acid	pH
Ala	6.3 ∼ 6.5	Val	6.4 ∼ 7.2
Leu	5.9 ∼ 6.0	Asp	6.0 ∼ 10.0
Arg	3.5 ∼ 8.9	Trp	5.6 ∼ 5.8
Pro	10.9 ∼ 11.5	Tyt	5.4 ∼ 5.8
Tri-Ala	4.9 ∼ 5.3	Phe	4.7 ∼ 4.9

## 3. Results

### 3.1. Experimental adsorption of amino acids by C_60_ or FNWs

The amino acids used are classified by the side chain. Ala, Val and Leu have an alkyl chain, Asp has a carboxyl group, Arg has an amino group and Phe, Tyr, Trp and Pro have an aromatic and an indole group, and a cyclic structure, respectively.

**Figure 2.**
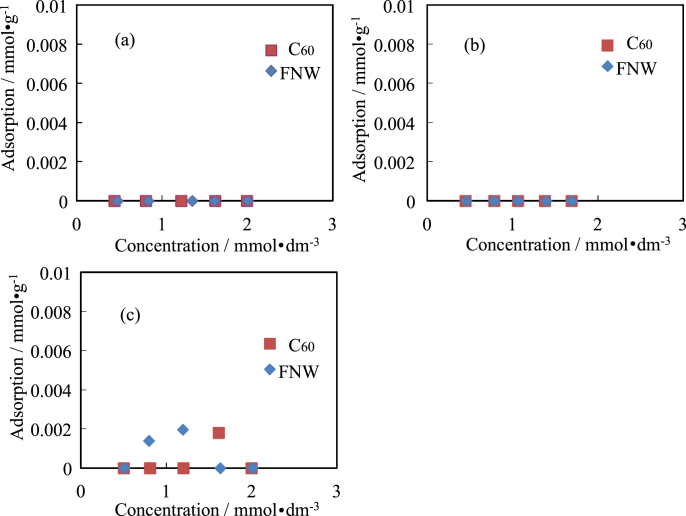
Isotherms for adsorption of Ala (a), Val (b) and Leu (c) by FNWs and C_60_.

**Figure 3.**
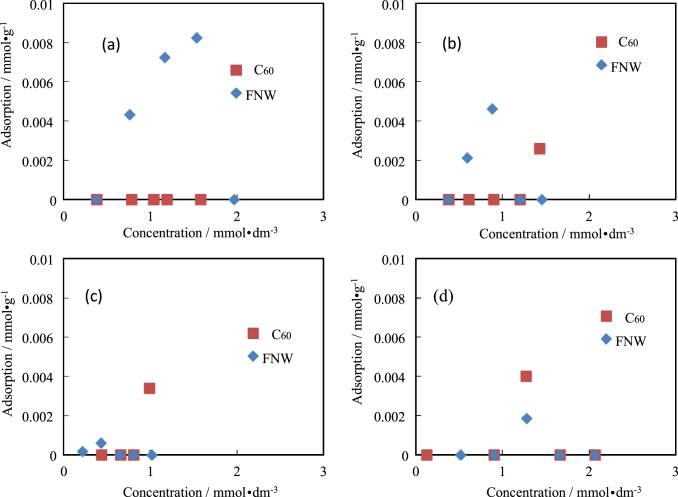
Isotherms for adsorption of Phe (a), Tyr (b), Trp (c) and Pro (d) by FNWs and C_60_.

**Figure 4.**
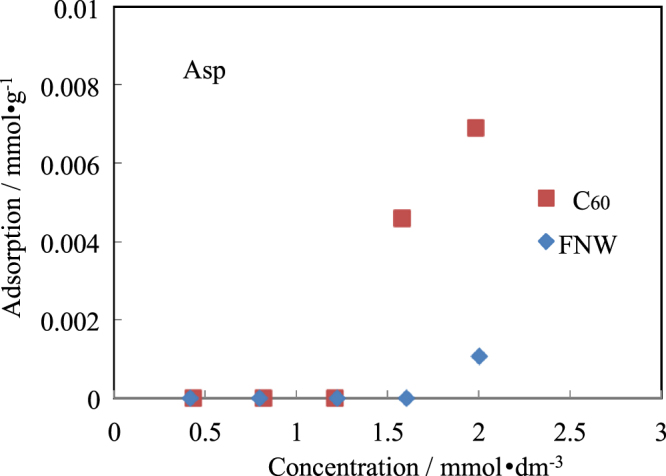
Isotherms for adsorption of Asp by FNWs and C_60_.

**Figure 5.**
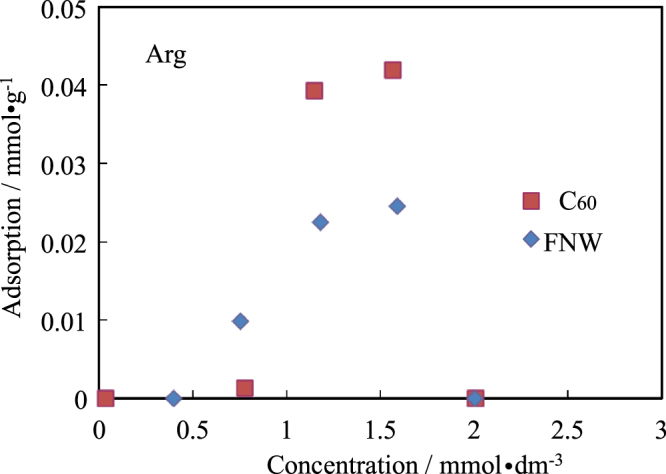
Isotherms for adsorption of Arg by FNWs and C_60_.

Isotherms for the adsorption of amino acids by C_60_ and FNWs are shown in figures 2–5. The side chains of Ala, Val and Leu were hydrophobic. Ala and Val were not adsorbed by C_60_ or FNWs. However, Leu could be adsorbed slightly by FNWs (max. 0.0020 mmol dm^−3^) and C_60_ (max. 0.0018 mmol dm^−3^) as shown in figure 2. Leu might be adsorbed by FNWs and C_60_ in effect on the amino acid’s side chain. However these extents of adsorption might be within the various errors. Ala, Val and Leu were seldom adsorbed by them. Amino acids, of which the side chain is an aromatic, an indole or of a cyclic structure, (e.g. Phe, Tyr, Trp and Pro) were also hardly adsorbed by C_60_ and FNWs. Phe might be adsorbed slightly by FNWs (figure 3(a)). In the adsorption of Trp and Pro, one experimental point shows the extent of adsorption. The point has some highly experimental uncertainties though the pH range is not so wide in the adsorption treatment (table 1). FNWs might adsorb Tyr. And Asp and Arg, which have a positive or a negative charge, respectively, were also not adsorbed by the C_60_ and FNWs (figures 4 and 5). In the adsorption treatment of Asp, the pH varied greatly (table 1). C_60_ preferentially adsorbs Asp in concentrated solutions with pH 2–8. Arg is adsorbed by FNWs and C_60_. Arg is adsorbed more strongly than other amino acids (see figures 2–5).

### 3.2. Experimental adsorption of oligo-Ala by C_60_ and FNWs

**Figure 6.**
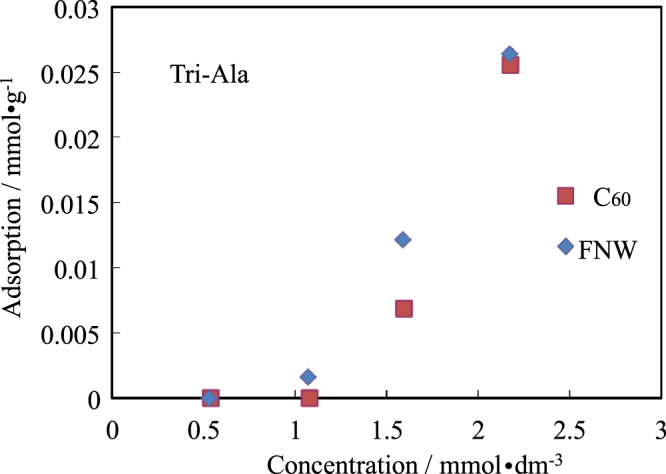
Isotherms for adsorption of Tri-D-Ala by FNWs and C_60_.

The isotherms for the adsorption of Tri-Ala are shown in figure 6. Tri-Ala can be adsorbed by C_60_ and FNWs. The charge of carboxyl and the amino group will not have a strong effect on the interaction of the side chain (methyl group) and C_60_ and FNWs.

## 4. Discussion

### 4.1. Interaction between amino acids and C_60_ and its derivatives

We used typical amino acids, which had aromatic, alkyl, carboxyl and amino groups with no sulfur or hydroxide groups in the side chains. Most amino acids will not be adsorbed by C_60_ and FNWs by virtue of physical and chemical interactions such as the electrostatic force, hydrogen bonding, and chemical reactions etc. Since Phe, Trp, Tyr and Pro have aromatic or cyclic side chains, we expected that the surfaces of C_60_ and FNWs would interact with the side chain of those amino acids. The amino or carboxyl group of the amino acid frame probably has a charge like –NH^3+^ or –COO^−^ in the solution. The charge of the amino acids would prevent interaction between amino acids and C_60_ or FNWs.

Although there have been hardly any experimental investigations into the adsorption of amino acids by C_60_ and FNWs, theoretical simulation groups have investigated the interaction between amino acids and C_60_ or C_80_ fullerene. De Leon *et al* showed the interaction between 20 bio-amino acids and C_60_ using theoretical simulation [5]. When we pay attention to the amino acids we used, the hydrogen of the frame of Ala, Asp, Phe or Pro was combined with C_60_. The distance is from 0.272 (Asp) to 0.442 (Ala) nm. In the case of Val, Arg and Trp, there is also another weak bond between the hydrogen of the side chain and C_60_. In addition, Leu and Tyr have three weak bonds with C_60_ involving the hydrogen of the amino acid frame and the side chain. The oxygen of the carboxyl group of Leu was combined with C_60_. On the other hand, the hydrogen of the amine group of the Tyr frame was combined with C_60_ as the weak bond. They also showed the free energy of dissociation to increase in the order Val < Phe < Pro < Asp < Ala < Trp < Tyr < Arg < Leu. In their results, the hydrogen of the frame of amino acids combined with C_60_ by a weak bond, while the side chain did not have a strong effect on the adsorption of amino acids and C_60_. The combination of 20 bio-amino acids with C_80_ fullerene was theoretically investigated by de Leon *et al* [6]. The free energies of dissociation of C_80_ were generally small as compared with those of C_60_. The hydrogen of the amino acid frame was connected with C_80_ as the hydrogen bond. Other hydrogen and/or oxygen did not affect the adsorption behavior of C_80_.

We did not observe significant adsorption of amino acids by C_60_ and FNWs, contrary to theoretical predictions by de Leon *et al* [5]. Since Arg and Tyr were adsorbed by C_60_ and FNWs, the adsorption of Arg and Tyr might affect the bonding. De Leon *et al* used an idealized model and neglected the charge of the amino and carboxyl groups of the frame such as –NH^3+^ and –COO^−^. When an amino acid dissolves in water, the amino group and/or carboxyl group has a positive and/or a negative charge. The reason why C_60_ did not adsorb amino acids in our experiment is mainly related to the charge. C_60_ prefers the hydrophobic interaction and will not prefer the hydrophilic materials in the adsorption behavior, that is to say that C_60_ dissolves in toluene but does not dissolve in water, generally. An organic molecule with a hydrophilic group such as the amino and carboxyl groups will hardly be adsorbed by the C_60_ surface.

### 4.2. Interaction between peptides or proteins and C_60_ and its derivatives

In the interaction between the side chains of the amino acids with C_60_ and FNWs, carboxyl and amino groups in the frame of the amino acid are mainly charged positively and/or negatively. If the charged carboxyl or amino group affects adsorption by C_60_ and FNWs, the carboxyl and amino groups of peptides might have less effect on the interaction of the side chain with C_60_ and FNWs, because the middle of the side chain of the peptide will not be close to the charged carboxyl or amino group. Figure 6 shows that Tri-Ala was adsorbed more by C_60_ and FNWs than the Ala monomer.

The adsorption of proteins by C_60_ was investigated [1, 23, 24]. It was demonstrated that small C_60_ aggregates could adsorb human and bovine serum albumins. The results were almost the same, such that the side chain of Tyr or Trp was adsorbed by the C_60_ aggregate. The structure of the albumin changed due to the adsorption by the C_60_ aggregate. In particular, an α-helix changed due to the interaction between C_60_ and the albumin. The side chains of Tyr (indole group) and Tyr (phenyl group) would react to the six-membered ring of C_60_ by the hydrophobic interaction and the structure around Trp and Tyr would be slightly changes by the C_60_. The concentrations of C_60_ and albumin are also important for the adsorption between them. The interaction between C_60_ and albumin was hindered at high and low concentrations of C_60_ in the constant albumin solution. In addition, although a C_60_ molecule did not affect the structure of albumin, a small C_60_ aggregate affected it [1]. Trp was not adsorbed by C_60_ and FNWs in this work. The charge of the carboxyl and amino groups have a strong effect on the adsorption of Trp. In the case of Tyr, its concentration might affect the adsorption behavior by C_60_ and FNW. And also, the concentration of other amino acids solutions might affect the adsorption behavior to C_60_ and FNW.

The interaction between graphene or single-wall carbon nanotubes (SWCNTs) and a protein was investigated by Zuo *et al* [8]. Trp and Tyr affected adsorption behavior. They also expected a similar adsorption mechanism, like hydrophobic interaction, between Trp or Tyr and graphene or SWCNTs.

## 5. Conclusions

The interactions of C_60_ and FNWs with amino acids were investigated. C_60_ and FNWs did not adsorb the mono-amino acids we used. Tri-Ala could be slightly adsorbed by C_60_ and FNWs. The mono-amino acids have a (positive or negative) charge. The chargeable carboxyl and amino groups of the frame of the amino acid would disturb the adsorption of the side chain of the amino acid. The side chain of methyl groups in the middle of Ala in Tri-Ala was slightly too far from both the carboxyl and the amino group of the Tri-Ala frame structure.

The interaction between protein and C_60_ showed that the aggregate of C_60_ could adsorb albumin. The concentration of C_60_ or protein affected the adsorption. The size of the C_60_ also had an effect on the adsorption of protein. C_60_ aggregates such as FNWs may interact with amino acids and their oligomers [1].

C_60_ and FNWs are very important nanomaterials for potential medical and pharmaceutical applications, yet their toxicity is not fully understood. Nanocarbon materials such as C_60_, SWCNTs and graphene might have detrimental effects when injected into the human body or individual cells [2]. Under normal conditions, when carbon nanomaterials are taken into the body, for example when food is contaminated by them, there are a few routes through which the carbon nanomaterials can enter a cell; by the affinity of cell proteins, passing through the cell’s wall and/or penetrating the cell’s wall, and so on. In this work, mono-amino acids hardly interacted with C_60_ and FNWs. It is expected that C_60_ and FNWs do not enter the cell to use the cell protein. The pH level is 7.35–7.45 in the human body, but it may differ in other forms of life. The pH range in this work could not be adjusted to the pH range of life. We will have to carry out further investigations into the adsorption of amino acids by C_60_ and FNWs depending on pH, including an electrolyte. Moreover, the adsorption of oligopeptides or polypeptides by C_60_ and FNWs might show different behaviors from that of albumin by C_60_ [1, 23, 24]. We will have to investigate the interaction between C_60_ or FNWs and some kind of peptides, and the relationship between concentration of C_60_ or FNWs, and mono-amino acids or polypeptides.
